# Momentarily narcissistic? Development of a short, state version of the Pathological Narcissism Inventory applicable in momentary assessment

**DOI:** 10.3389/fpsyg.2022.992271

**Published:** 2022-10-26

**Authors:** Márton Engyel, Naomi M.P. de Ruiter, Róbert Urbán

**Affiliations:** ^1^Doctoral School of Psychology, ELTE Eötvös Loránd University, Budapest, Hungary; ^2^Institute of Psychology, ELTE Eötvös Loránd University, Budapest, Hungary; ^3^University College Groningen, University of Groningen, Groningen, Netherlands

**Keywords:** narcissism, grandiosity, vulnerability, momentary measurement, narcissistic states, confirmatory factor analysis, multilevel analysis, experience sampling method

## Abstract

**Background:**

Narcissism viewed as a personality process rather than a stable trait explains narcissistic functioning as a tool for maintaining a positive self-view. Studying narcissism therefore needs adequate momentary measures for collecting higher frequency longitudinal data in experience sampling method (ESM) studies. In this study, a shorter version of the Pathological Narcisissm Inventory is offered to measure vulnerable and grandiose narcissistic states, applicable in momentary assessment.

**Methods:**

The measurement tool was tested in three samples. First, we assessed the factor structure and associations with other contemporary measures of narcissism in a cross-sectional design on one English speaking (*n* = 319) and one Hungarian sample (*n* = 236). Second, we conducted a five-day long experience sampling method study with a total of 15 measurement points (*n* = 123).

**Results:**

Based on structural equation modelling and multilevel analyses, the results suggest that the measure has adequate psychometric properties in both the within and between subject levels as well as acceptable convergent and discriminant validity.

**Conclusions:**

The Pathological Narcissism Inventory – State Version (PNI-S) can be a useful tool in momentary data collection enabling the examination of personality processes behind narcissistic functioning.

## Introduction

In recent years the study of narcissism has expanded substantially, however some important questions are still left open (Miller et al., 2021). Researchers agree that narcissistic behaviours can be categorised into at least two broader subtypes, namely *grandiose* and *vulnerable* narcissism (Wink, 1996). Individuals with grandiose narcissistic traits are described as arrogant, exploitative, and entitled (Cain et al., 2008), and they often engage in self-aggrandisement and self-promotion while also striving for a sense of uniqueness (Zeigler-Hill et al., 2008; Miller et al., 2017). By contrast, vulnerable narcissism is characterised by defensive social withdrawal, self-inhibition, and substantial reliance on the approval of others for feelings of self-worth (Cain et al., 2008; Zeigler-Hill et al., 2008). At the same time, these individuals also hold grandiose expectations of oneself and others (Wink, 1996) and they tend to be overly sensitive to the feelings of embarrassment and shame (Edershile et al., 2019).

Recently, two hierarchical models were proposed to integrate the seemingly incoherent nature of narcissistic grandiosity and vulnerability (Wright and Edershile, 2018; Miller et al., 2021). Krizan and Herlache (2018) offered the Narcissism Spectrum Model (NSM) placing self-importance and entitlement on the core of narcissistic functioning. In their model narcissistic vulnerability would be a phenotypic manifestation of the reactive orientation system characterised by avoidance while narcissistic grandiosity is reflecting the approach-dominant orientation system (Gray and McNaughton, 2000). Miller et al. (2016) on the other hand applied the five-factor model of personality and argued that antagonism with features like exploitativeness, entitlement or reactive anger serve as the core of narcissistic functioning while vulnerability can be understood through neuroticism, and grandiosity through agentic traits of extraversion. These two hierarchical solutions are similar in nature highlighting general narcissistic tendencies, while also differentiating functionally divergent manifestations (Wright and Edershile, 2018).

Besides these hierarchical models another recent conceptualization of narcissism is aiming to separate two social strategies of how narcissistic individuals aim to maintain their grandiose self. In the Narcissistic Admiration and Rivalry Concept (NARC; Back et al., 2013; Back, 2018) the pathway of narcissistic admiration serves as an assertive self-enhancement strategy to approach positive social incentives (e.g., grandiose fantasies, striving for uniqueness), while narcissistic rivalry aims to prevent social failure by protecting the self in an antagonistic manner (e.g., devaluation of others, striving for supremacy), although these strategies can be considered as agentic or antagonistic behaviour patterns from the viewpoint of the five-factor model (Back, 2018). Similarly to the hierarchical conceptualizations, the NARC model also identifies a general, overarching goal behind distinct narcissistic behaviours, namely the maintenance of the grandiose view of the self.

Vulnerable and grandiose narcissism has substantially different nomological networks (Miller et al., 2011). Regarding personality traits, grandiosity is positively associated with extraversion (e.g., facets assertiveness and excitement seeking) while negatively with agreeableness (e.g., facets compliance and modesty). Vulnerability on the other hand is in strong positive association with neuroticism (e.g., facets depression and angry hostility) and a moderate negative correlation with agreeableness.

These differences also exist in other important aspects of personality functioning for example implicit and explicit self-esteem (Miller et al., 2011; Mota et al., 2019), or most indicators of psychological well-being. Grandiose narcissism is in a moderate positive association with subjective well-being (Egan et al., 2014; Czarna et al., 2018), life satisfaction (Sedikides et al., 2004) frequent experiences of positive emotions (Rhodewalt et al., 1998) or self-esteem (Miller et al., 2011). In contrast, vulnerable narcissism is negatively associated with factors of eudaimonic well-being (Kaufman et al., 2018) and positively to negative emotionality, depression and low self-esteem (Miller et al., 2011, 2017).

Measurement of narcissism was also subject to clarification recently, as several measures of grandiosity and vulnerability emerged over the years, and different underlying conceptualizations made the synthetisation of results difficult (Miller et al., 2021). Narcissism measures can therefore be categorized by measuring either vulnerable or grandiose narcissism or both. Currently the most widespread measure of grandiose narcissism is the Narcissistic Personality Inventory (NPI; Raskin and Terry, 1988; Cain et al., 2008), although several other psychometrically sound measures were introduced in recent years (e.g., the Narcissistic Grandiosity Scale NGS; Rosenthal et al., 2007). Separate measurement of vulnerable narcissism is also supported (e.g., the Maladaptive Covert Narcissism Scale; Cheek et al., 2013 or the Narcissistic Vulnerability Scale; NVS; Crowe et al., 2018), moreover several measures are assessing the two dimensions as facets of one instrument e.g., the Pathological Narcissism Inventory (PNI; Pincus et al., 2009); or the Five-Factor Narcissism Inventory (FFNI; Glover et al., 2012).

With recent hierarchical models conceptualizing vulnerability and grandiosity as personality moderated expressions of the same core (Wright and Edershile, 2018), a three-dimensional approach has also been applied in evaluating different measurement methods. The role of differentiating entitlement, grandiosity and vulnerability is mainly to enable a more unified approach in measuring these constructs with several different tools (Wright and Edershile, 2018). According to this concept, different underlying factors are captured more precisely by different measurement tools. For example, the NGS (Rosenthal et al., 2007) is rather measuring exhibitionism than entitlement, while the grandiosity and vulnerability factors of the PNI (Pincus et al., 2009) both capture the “entitlement core” of narcissism (for more details see Wright and Edershile, 2018). Although the focus of this scale is closer to vulnerability (Krizan and Herlache, 2018; Crowe et al., 2019) and maladaptive manifestations of narcissism (Pincus et al., 2009), central elements of grandiosity are also captured sufficiently especially when applying a multivariate approach (Edershile et al., 2019). In this study we used the PNI for developing a state level measure of narcissistic functioning mostly because it captures not only exhibitionism and vulnerability but also entitlement and the antagonistic core of narcissism in both factors. The dominant use of the above scales reflects the similarly dominant view that narcissism is a stable trait (for a review, see Campbell and Miller, 2011), however how these descriptive behaviours converge at the individual level and in real-time is less well understood (Wright and Edershile, 2018; Edershile and Wright, 2021). Trait level measurement on the other hand is also subject to several biases coming from aggregation of experiences or the lack of reliable processes of memory recollection (for a review, see Hektner et al., 2007). Moreover, from a methodological point of view narcissism is mainly studied in between-subject settings, which offers insights into structural and dispositional differences between people, however internal personality processes can be examined rather on the within-subject level (Bolger et al., 2003) and with intensive longitudinal data (Wright and Edershile, 2018).

Zooming in on the narcissistic *process* (which we refer to as including actions, feelings, and thoughts) that characterise narcissistic traits can help in understanding why and when this process operates and in which circumstances does it result in positive versus negative subjective experiences. Recent models of narcissism are beginning to conceptualise narcissism as a dynamic self-regulatory process (Giacomin, 2016) in which the narcissistic process is used to maintain positive self-views (Morf and Rhodewalt, 2001; for a review, see Giacomin, 2016). This growing focus on the understanding of within-individual processes underlies the need to measure narcissistic actions, feelings, and thoughts from moment to moment enabling us to observe individual trajectories of momentarily narcissistic tendencies and how the stability of this process emerges over time (i.e., the stability that characterises traits). Furthermore, focusing on these trajectories of behaviours would allow us to understand how vulnerable and grandiose narcissistic tendencies are temporally associated (Edershile et al., 2019). This is crucial because vulnerable and grandiose behaviours correlate weakly on the trait level (Miller et al., 2010), they have different nomological networks (Miller et al., 2011), although both of them can occur within the same individual present at different times or situations according to clinical theories and observations (Wink, 1996; Edershile et al., 2019). Furthermore, a recent study by Jauk and his colleagues (2021) emphasized a nonlinear association between grandiose and vulnerable traits indicating, that at higher levels of grandiosity vulnerability increases, indirectly reflecting the possible effects of state changes.

With a growing interest in the processes that underlie narcissistic functioning (Geukes et al., 2017; Edershile et al., 2019), there are different approaches for measuring grandiose and vulnerable narcissism as momentary assessments (i.e., states). Giacomin (2016), for example, mainly used trait measures adapted to capture states by changing the instructions such that participants were asked to reflect on their *current* states. This, however, might not be ideal because most items refer to general or aggregated personal qualities. Nevertheless, this step toward a state measure revealed moderate fluctuations across contexts.

In other studies, the adjective-based Narcissistic Grandiosity Scale (NGS) and the Narcissistic Vulnerability Scale (NVS) were used as state measurement, (e.g., Edershile et al., 2019; Edershile and Wright, 2021) where participants needed to decide how they feel about themselves at the moment. Originally, the NGS consists of 16 adjectives (e.g., glorious, prestigious) while the NVS offers 11 (e.g., underappreciated, insecure), although in most studies using intensive longitudinal data a shorter version of the scales with 4–4 items demonstrated good psychometric properties (Crowe et al., 2016, 2018; Edershile et al., 2019).

Narcissism viewed from the dynamic self-regulatory perspective (Morf and Rhodewalt, 2001) can be understood as a set of feelings, actions and behaviours that the individual tends to use to maintain positive self-views. Therefore, limiting the scope of study to the self-related feelings, might limit our ability to evaluate every aspect connected to narcissistic states when they are not accompanied by stronger feelings.

The state-like measures of narcissism demonstrate an important step toward capturing the dynamics of narcissistic behaviours. However, to collect higher-frequency longitudinal data in experience sampling method studies (Hektner et al., 2007), it is crucial that researchers use the shortest assessment possible while ensuring that validity is protected. Furthermore, researchers need to minimise the chance, that a time-consuming assessment might interfere with the process they wish to study.

In this study we aimed to develop a tool for assessing narcissistic states, which is necessary to study how and when the narcissistic process emerges. We assessed the psychometric properties of a state measure of narcissism focusing on momentary narcissistic behaviours. It consists of seven items, making it shorter than the previous measures used (NGS and NVS; Edershile et al., 2019), assessing both vulnerable (four items) and grandiose (three items) narcissistic functioning. On two different samples we assessed the convergent and discriminant validity of the measure with the currently used trait measurements of narcissism and self-esteem. On the third sample, we tested its within-and between-subject level associations using structural equation modelling and multilevel analyses, based on a five-days long experience sampling method setting. We hypothesized that state-level vulnerable narcissism would be in positive relationship with other current measures of vulnerable narcissism and in negative relationship with self-esteem and psychological well-being (Miller et al., 2018). We also expected that grandiose narcissistic states would be in positive correlation with other contemporary measures of grandiosity, and in positive correlation with self-esteem and psychological well-being (Miller et al., 2011; Hyatt et al., 2018). In this research we selected the criterion variables based on their consistent and well-established associations with grandiose and vulnerable narcissism in previous studies (e.g., Miller et al., 2011; Aghababaei and Błachnio, 2015; Kaufman et al., 2018).

Lastly, we expected weak but positive relationship between grandiose and vulnerable states on the between-subject level representing the entitlement core of the distinct manifestations. In contrast, on the within-subject level we expected negative relationship between grandiose and vulnerable states as with context-dependent fluctuations grandiosity and vulnerability were not expected to be present at the same time (Wink, 1996; Edershile et al., 2019).

## Materials and methods

### Participants and procedure

In this study three different samples were administered in different languages and with different settings.

In Sample 1 (S1), participants were recruited from a pool of fluent speaking first-year international students from a large Dutch university who participated in exchange for course credits. The measures were administered in English. Participants registered for the study through a secure online portal from which they were redirected to the online surveys. A total of 319 participants (73% female; mean age = 20.18; *SD* = 2.31) filled out the measures. The study was approved by the Ethical Committee of Psychology, University of Groningen (registration number: 18102-S).

In Sample 2 (S2) participants were recruited from a pool of students from a large Hungarian university who participated in exchange for course credits. The measures were administered in Hungarian. Participants registered for the study through a secure online portal from which they were redirected to the online surveys. A total of 236 participants (75% female; mean age = 22.10; *SD* = 3.91) filled out the measures.

In Sample 3 (S3), participants were recruited from a large university in Hungary who participated in exchange for course credits. The measures were administered in Hungarian. A total of 128 participants completed the first wave of the study in which they filled out trait measures and provided their mobile phone numbers. A total of 123 participants completed the second part of the study with at least 80% fill-out rate (66.4% female; mean age = 21.84; *SD* = 3.53) in which participants had to fill out three measurements per day for five consecutive days. The five-day period started on a Tuesday and lasted until Saturday. Participants were sent a short message with the current questionnaire’s link, which could be easily filled out on an Android or iOS smartphone. Three momentary questionnaires were sent out at a random time within three separate time frames: from 8:00 to 11:00, from 12:00 to 15:00, and from 16:00 to 19:00. Questionnaires were distributed at least 2 h after the previous measurement. The study containing Sample 2 and Sample 3 was approved by the Ethical Committee of Eötvös Loránd University, Hungary (registration number: 2018/229).

### Measures

#### Narcissistic personality inventory (NPI-40)

The Likert version of the original English version of the NPI-40 (Raskin and Terry, 1988) was administered in Sample 1, which presents only the original 40 narcissistic items. Participants stated how much each item described them on a scale from one to five. This response format of the NPI is recently gaining popularity (Wetzel et al., 2016; Miller et al., 2018; Engyel et al., 2022). The NPI was administered in Samples 1 and Sample 3.

#### Pathological narcissism inventory (PNI)

The PNI (Pincus et al., 2009) assesses seven factors of both vulnerable and grandiose narcissism. A factor structure was proposed of two higher-order dimensions (Wright et al., 2010): Exploitativeness, Self-Sacrificing, Self-Enhancement, and Grandiose Fantasies together form the grandiosity factor while Contingent Self-Esteem, Hiding the Self, Devaluing, and Entitlement Rage form the vulnerability factor. Items are rated from “Not at all like me” as zero to “Very much like me” as five. The PNI was administered in Sample 1 and Sample 3.

#### Narcissistic grandiosity scale (NGS)

The NGS (Rosenthal et al., 2007; Crowe et al., 2016) is an adjective scale that contains 16 items. Participants are presented with the 16 adjectives (e.g., Glorious, Prestigious) and asked to rate how much these adjectives describe themselves. The scale has been recently demonstrated to have good psychometric properties (Crowe et al., 2016). The NGS was administered in Sample 1.

#### Narcissistic vulnerability scale (NVS)

The NVS (Crowe et al., 2018) is also an adjective-based measure similar to the NGS with 11 items such as *Underappreciated*, *Insecure*, and *Fragile*. Participants are asked to state how much these adjectives describe them on a scale from one to seven. The NVS was administered in Sample 1.

#### Maladaptive covert narcissism scale (MCNS)

The MCNS (Cheek et al., 2013) is a Likert-type measure assessing hypersensitive or vulnerable narcissism. It consists of 23 items with answer options ranging from one to five. This scale is considered as a significantly improved version (Cheek et al., 2013) of the original Hypersensitive Narcissism Scale (Hendin and Cheek, 1997). The MCNS was administered in Sample 1 and Sample 3.

#### Self-esteem

Self-esteem was assessed using the Rosenberg Self-esteem Scale (RSES; Rosenberg, 1965), a widely used 10-item scale capturing global self-esteem. Participants respond to questions (e.g., “I am able to do things as well as most other people”) on a 4-point Likert scale ranging from zero – “strongly disagree” to three – “strongly agree.” The measure consists of five reversed items. The RSES was administered in Sample 1 and Sample 3.

#### State self-esteem

State self-esteem was assessed in Sample 3 as a momentary assessment. We used one positive and one negative statement from the Rosenberg (1965) self-esteem scale (RSES), changing the framing to capture momentary states. The two items were “Right now, I feel that I cannot be proud of anything” and “Right now, I feel that I have a number of good qualities.”

#### Eudaimonic well-being

To measure eudaimonic well-being the Hungarian version of the Ryff and Keyes (1995) Well-Being Scale was used which assesses Ryff’s (1989) original six factors of eudaimonic well-being with three items for each factor: purpose in life, autonomy, personal growth, self-acceptance, positive relations and environmental mastery. The measure offered good internal consistency in our sample (*α* = 0.86). This scale was administered in Sample 3.

#### Pathological narcissism inventory – state version (PNI-S)

We aimed to assess both vulnerable and grandiose narcissism as states. Based on our preliminary analysis, using a combination of data-driven psychometric and judgmental content-related considerations (for a review, see Kruyen et al., 2013) we used one item from each of the seven subfactors of the Pathological Narcissism Inventory (PNI; Pincus et al., 2009). Items were considered appropriate if they represented the latent factors well based on the factor loadings in the original study of Pincus et al. (2009). First, items with factor loadings higher than 0.7 were selected in each subfactor of the PNI, following the recommendation of Tabachnick et al. (2007). Second, items were selected if they could be understood as a current state of mind (e.g., “I often fantasize about having a huge impact on the world around me.”) rather than just aggregated personal qualities. Selected items were judged by the members of our research team regarding their ability to capture the content of the PNI subscale well. Last, the highest rated items were tailored to measure the momentary experience of the participant (e.g., “Right now, I am having fantasies of having a huge impact on the world around me.”). The phrase “Right now,” was included in all items to avoid possible effects of skipping the measure instructions and to help participants focusing on their current state of mind. The vulnerable narcissistic state was therefore assessed with four items while the grandiose state was captured with three items. Items of the PNI-S are presented in Table 1.

**Table 1:** Items of the PNI-S with the related facets from the original PNI and an exploratory factor analysis of the PNI-S in the international and Hungarian samples.

		International students Sample 1 (*n* = 319)		Hungarian students Sample 2 (*n* = 236)	
Facets of the original PNI	Items of the PNI-S	Vulnerable narcissistic state *α* = 0.76	Grandiose narcissistic state *α* = 0.44	Vulnerable narcissistic state *α* = 0.74	Grandiose narcissistic state *α* = 0.51
Contingent self-esteem	Right now, I am feeling bad about myself because other people do not notice me.	**0.80**	0.06	**0.81**	−0.03
Entitlement rage	Right now, I am feeling annoyed because others are not interested in what I am saying or doing.	**0.76**	0.13	**0.70**	0.07
Devaluing	Right now, I am avoiding people, because I am concerned, that they will disappoint me.	**0.54**	0.15	**0.62**	−0.10
Hiding the self	Right now, I am hiding my needs for fear that others will see me as needy and dependent.	**0.57**	−0.03	**0.51**	0.11
Exploitative	Right now, I feel that I can make anyone believe anything I want them to.	0.08	**0.70**	0.07	**0.55**
Self-sacrificing self-enhancement	Right now, I feel that I am important because others can rely on me.	−0.04	**0.31**	−0.34	**0.50**
Grandiose fantasy	Right now, I am having fantasies of having a huge impact on the world around me.	0.25	**0.58**	0.07	**0.47**
	Eigenvalues	2.47	1.49	2.45	1.49
	Explained Variance	35.2%	21.3%	35.1%	21.2%

The correlations between factors were *r* = 0.11 in the international student sample (Sample 1) and *r* = −0.05 in the Hungarian sample (Sample 2). PNI-S, Pathological Narcissism Inventory State Version; PNI, Pathological Narcissism Inventory. Values in boldface indicate the dominant loading.

The Hungarian translation of the items was carried out following the guidelines of the standard test-adaptation procedure: first, two members of the research group translated the items separately, then a back-translation was applied. The internal consistency of the subscales is presented in both the English and Hungarian measures in Table 1. The subscale measuring vulnerable narcissistic states offers acceptable reliability in both samples, however the α for grandiose states remains moderate, due to the limited number of items, and due to the fact, that two items out of the three had substantially higher factor loadings in the scale. Moreover, coefficient alpha can be underestimated when tests are short (Cronbach, 1951; Hoekstra et al., 2019) and ranges of acceptable alpha is also lower when measures have fewer than 10 items (Pallant, 2011).

### Statistical analysis plan

#### Descriptive statistics and intraclass correlation coefficients

To compare the nomological network of PNI-S grandiosity and vulnerability factors with other contemporary measures of narcissism and other correlates intraclass correlation coefficients (ICC) were calculated. ICC values above 0.8 are regarded as signs of good reliability (Koo and Li, 2016; Liljequist et al., 2019). First, PNI-S factors were compared to the original PNI grandiosity and vulnerability factors. Second, other grandiosity measures, vulnerability measures and external correlates were used excluding the PNI due to the considerable overlap between the state and trait constructs.

#### Exploratory factor analysis

First, we applied an exploratory factor analysis (EFA) using SPSS version 25 on the data to explore the factor structure of the 7-item long measure. We applied the Maximum Likelihood method for factor extraction, with a direct oblimin rotation on both the English version (S1), both the Hungarian version (S2) of the measure. Factors with eigenvalues greater than 1 were extracted.

#### Confirmatory factor analysis with covariates

Second, we performed confirmatory factor analyses (CFA) using Mplus 8.3 (Muthén and Muthén, 1998–2015). In a CFA, a satisfactory degree of fit requires the comparative fit index (CFI) and the Tucker-Lewis Index (TLI) to be close to 0.95, and the model should be rejected when these indices are less than 0.90 (Brown, 2006). The next fit index was the root mean square error of approximation (RMSEA). RMSEA below 0.05 indicates excellent fit, a value around 0.08 indicates adequate fit, and a value above 0.10 indicates poor fit (Hu and Bentler, 1999).

We applied a CFA on S1 to assess the associations of the two-factor measurement model of grandiose and vulnerable states with other measures of narcissism and personality functioning using a CFA with covariates analysis.

Furthermore, we conducted a multilevel CFA on the data from Sample 3 to differentiate the within-subject and the between-subject level in our analysis (Muthén and Muthén, 1998–2015).

#### Within-subject level analysis

We also tested the intra-individual associations of the state measures in an experience sampling method setting on Sample 3. We used 15 measurement points, and therefore the standard methods for evaluating the association could not be used without violating the assumption of independence of observations.

Therefore, the present study applied the dynamic structural equation modelling framework (DSEM) using Mplus (McNeish and Hamaker, 2019), which enables the integration of both SEM models and time-series analysis (Asparouhov et al., 2018). Our data contained information on two levels, 15 measurement points were nested within a person. Therefore, using multilevel modelling, the within-subject level dynamics of narcissistic states can be separated from the associations captured in the between-subject level.

**Figure 1:**
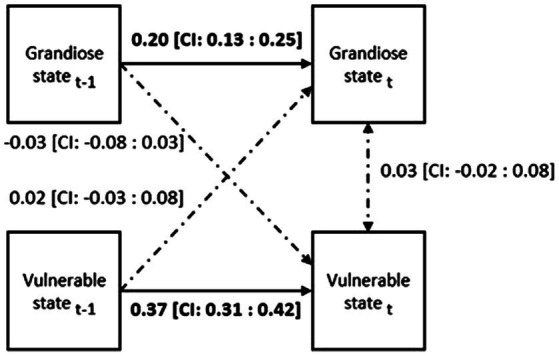
The stability of state constructs: Within level analysis of the multilevel cross-lagged autoregressive model. Note: All estimates are standardized, bold estimates are significant on *p*<0.001. CI: 95% credibility interval.

Our model is presented in Figure 1. As we had two outcome variables collected at each measurement, besides the autoregression (when a variable from the previous time point predicts the same variable in a following time point) we were also interested in the cross-lagged associations between them (meaning the effect of the first variable collected in a time point on the second variable in the preceding time point), we used a multilevel cross-lagged vector autoregressive model [multilevel VAR (1)]. These autoregressive relationships are also good measures for examining construct stability (Hamaker et al., 2015), which was one of our main aims in this study.

To capture easily comparable results, we used standardized estimates in our analysis. Following the recommendations of Schuurman et al. (2016) we used within-subject standardization. The process was the following: first we standardized the regression coefficients for each person separately based on their within-subject variances. On the between-person level, standardization was based on the between person variances (McNeish and Hamaker, 2019). As our data collection technique was based on unequally spaced measurements the lagged effects of the model had to be corrected due to the time elapsed between the evening and morning data collection. Therefore, following the recommendation of Asparouhov et al. (2018) we used a 6 h interval as a baseline in our analyses.

#### Associations on the between-subject level

Regarding the associations of the PNI-S with other criterion variables in the momentary assessment, first we used repeated-measures correlation (Bakdash and Marusich, 2017) with the R package “Rmcorr” (R Core Team, 2017) to evaluate the association between vulnerable and grandiose states of narcissism and state self-esteem. Repeated-measures correlation also eliminates the problem of ergodicity between the different levels of analysis (Molenaar, 2004).

Lastly, we averaged all the momentary measurement points of narcissistic states and tested the between-subject level associations of the averaged scores with other contemporary measures of narcissism and criterion variables on Sample 3 using multiple regression. Grandiose and vulnerable states were used simultaneously as predictors to account for the unique variance each one explains.

## Results

### Descriptive statistics and intraclass correlation coefficients

Descriptive statistics and a correlation table on all study variables is reported in Table 2. The nomological networks of PNI-S grandiosity and vulnerability factors were compared using intraclass correlation coefficients on Sample 1. The following measures were used in comparison: Narcissistic Personality Inventory (NPI; Raskin and Terry, 1988), Narcissistic Grandiosity Scale (NGS; Crowe et al., 2016), Maladaptive Covert Narcissism Scale (MCNS; Cheek et al., 2013), Narcissistic Vulnerability Scale (NVS; Crowe et al., 2018), Rosenberg Self-esteem Scale (Rosenberg, 1965) and state self-esteem. Our results suggest that both the grandiosity (ICC ranges from 0.835 to 0.844) both the vulnerability factor (ICC ranges from 0.968 to 0.978) have similar associations with other measures of narcissism and external correlates. Furthermore, PNI-S factors were also compared to the original factors of the PNI. The ICC was high between vulnerability factors (ICC = 0.965) while moderate between grandiosity factors (ICC = 0.518). The most substantial difference in the associations of the PNI-S and PNI grandiosity factors were with measures of narcissistic vulnerability (NVS and MCNS) indicating, that the original PNI grandiosity factor also measures aspects of vulnerability, while the PNI-S grandiosity factor does not.

**Table 2:** Descriptive statistics, internal consistency, and associations of all study variables (Sample 1).

International students, Sample 1 (*n* = 319)													
	***M***	***SD***	***α***	PNI-S-G	PNI-S-V	PNI Total	PNI Gran.	PNI Vuln.	NPI	NGS	MCNS	NVS	SE
PNI-S-G	123.7	58.9	0.44										
PNI-S-V	113.8	83.8	0.76	0.127^*^									
PNI Total	170.8	37.2	0.94	0.291^**^	0.605^**^								
PNI Grandiosity	63.8	13.9	0.85	0.504^**^	0.254^**^	0.768^**^							
PNI Vulnerability	106.9	28.0	0.90	0.136^*^	0.678^**^	0.948^**^	0.524^**^						
NPI	110.9	21.1	0.91	0.552^**^	0.036	0.386^**^	0.576^**^	0.227^**^					
NGS	40.9	17.0	0.94	0.468^**^	−0.058	0.179^**^	0.336^**^	0.071	0.671^**^				
MCNS	63.2	13.2	0.85	0.079	0.579^**^	0.753^**^	0.439^**^	0.783^**^	0.198^**^	0.045			
NVS	30.0	11.6	0.87	0.023	0.645^**^	0.559^**^	0.261^**^	0.614^**^	0.021	0.000	0.614^**^		
SE	28.1	5.5	0.88	0.189^**^	−0.439^**^	−0.396^**^	−0.102	−0.476^**^	0.308^**^	0.401^**^	−0.512^**^	−0.560^**^	
State SE	14.1	3.8	0.89	0.144^**^	−0.414^**^	−0.311^**^	−0.077	−0.376^**^	0.222^**^	0.329^**^	−0.413^**^	−0.632^**^	0.745^**^

PNI-S-G, Pathological Narcissism Inventory State Version, Grandiosity Factor; PNI-S-V, Pathological Narcissism Inventory State Version, Vulnerability Factor; PNI Total, Pathological Narcissism Inventory Total Score; PNI Grandiosity, Pathological Narcissism Inventory Grandiosity Factor; PNI Vulnerability, Pathological Narcissism Inventory Vulnerability Factor; NPI, Narcissistic Personality Inventory; NGS, Narcissistic Grandiosity Scale; MCNS, Maladaptive Covert Narcissism Scale; NVS, Narcissistic Vulnerability Scale; SE, Self-esteem; State SE, State Self-esteem.

* *p* < 0.05 and

** *p* < 0.05.

### Exploratory factor analysis

An EFA was applied on the 7-item long measure both using the English (S1) and the Hungarian version (S2) of the test. Results indicate that the seven items form two distinct factors in both samples, factor loadings ranging from 0.31 to 0.81 (for the details, see Table 3).

**Table 3:** Comparing the nomological networks of the PNI-S factors with intraclass correlation coefficients (ICC) in Sample 1.

	PNI-S grandiosity	PNI-S vulnerability
	Intraclass correlation coefficient (ICC)	
PNI grandiosity factor	0.518	
NPI	0.835	
NGS	0.844	
PNI vulnerability factor		0.965
MCNS		0.978
NVS		0.968

PNI-S, Pathological Narcissism Inventory State Version; PNI, Pathological Narcissism Inventory; NPI, Narcissistic Personality Inventory; NGS, Narcissistic Grandiosity Scale; MCNS, Maladaptive Covert Narcissism Scale; NVS, Narcissistic Vulnerability Scale.

### Confirmatory factor analysis with covariates

In order to perform CFA with covariates analysis, we estimated the model fit of the two-factor measurement model of *PNI-S* on Sample 1. The results supported that the two-factor solution offers acceptable fit indices according to current traditions (RMSEA = 0.058; CFI = 0.961; TLI = 0.937; *χ*^2^ = 374.6; *df* = 21; *p* < 0.001). Standardised factor loadings ranged from 0.53 to 0.79 for the vulnerability factor and 0.23 to 0.81 for the grandiosity factor. Grandiosity and vulnerability factors were moderately positively associated (0.28). The factor loadings are presented in the Supplementary Material.

To examine the convergent and discriminant validity of the PNI-S, we applied a series of confirmatory factor analyses with covariates. The results are presented in Table 4. The grandiosity factor had a medium-strong positive relationship with the other standard measures of grandiose narcissism, but it was mostly unrelated to vulnerability measures. The vulnerability factor, on the other hand, was positively related to other measures of narcissistic vulnerability. Self-esteem and state self-esteem both had medium negative associations with the vulnerability factor while the grandiosity factor was not significantly related to any measures of self-esteem.

**Table 4:** Associations of the PNI-S in Sample 1: confirmatory factor analysis with covariates.

	Vulnerable narcissistic state	Grandiose narcissistic state	Difference p*
Narcissistic Personality Inventory (NPI)	0.05	**0.75**	<0.01
Pathological Narcissism Inventory (PNI)	**0.66**	**0.43**	<0.01
PNI Grandiosity factors	**0.27**	**0.68**	<0.01
PNI Vulnerability factors	**0.75**	**0.24**	<0.01
Narcissistic Vulnerability Scale (NVS)	**0.72**	**0.18**	<0.01
Narcissistic Grandiosity Scale (NGS)	−0.05	**0.61**	<0.01
Maladaptive Covert Narcissism Scale (MCNS)	**0.63**	**0.20**	<0.01
Rosenberg Self-Esteem Scale (RSES)	**−0.46**	0.15	<0.01
State Self-Esteem (SSE)	**−0.44**	0.08	<0.01
Gender	0.02	**−0.17**	0.20

*N* = 319. Standardised coefficients. Boldfaced scores are significant at least *p* < 0.05. Each covariate is regressed separately to avoid the multicollinearity of covariates.

* Wald-test was used in comparison of *β*s.

### Multilevel confirmatory factor analysis

The multilevel CFA used 1741 observations and offered acceptable fit indices according to current traditions (RMSEA = 0.035; CFI = 0.938; TLI = 0.900; *χ*^2^ = 960.2; *df* = 42; *p* < 0.001; SRMR_within-subject_ = 0.035; SRMR_between-subject_ = 0.093). Standardized factor loadings and intraclass correlations are presented on Table 5.

**Table 5:** Standardised factor loadings and intraclass correlations of the multilevel CFA.

Item	Intraclass correlation	Within-level factor loadings		Between-level factor loadings	
		Vulnerable state	Grandiose state	Vulnerable state	Grandiose state
SV* item 1	0.49	**0.67**		**0.99**	
SV* item 2	0.57	**0.44**		**0.71**	
SV* item 3	0.41	**0.60**		**0.92**	
SV* item 4	0.46	**0.56**		**0.82**	
SG** item 1	0.64		**0.52**		**0.71**
SG** item 2	0.47		**0.61**		**0.60**
SG** item 3	0.60		**0.48**		**0.80**

* State vulnerability item.

** State grandiosity item. Standardised coefficients. Boldfaced scores are significant at least *p* < 0.05.

On the within-subject level the association between the grandiosity and vulnerability factors is weakly negative (*r* = −0.32). On the between subject level however, the association between the two factors is weakly positive (*r* = 0.26).

### Measurement invariance

Measurement invariance was tested between the original English version of the measure and the Hungarian translation. The configural and the metric model were significantly different from each other (*χ*^2^ = 14.23, *df* = 5; *p* = 0.014), while the differences in the fit indices (configural model: *χ*^2^ = 49.44, CFI = 0.958, RMSEA = 0.062, CI [0.036–0.088]; metric model: *χ*^2^ = 71.65, CFI = 0.928, RMSEA = 0.069, CI [0.048–0.090]) were mixed. Following the recommendations of Chen (2007) whether sample sizes are adequate (total *N* > 300) measurement invariance between the models can be assumed if changes in the CFI measure is less than –0.01 supplemented by a change in the RMSEA measure less than 0.01. Between the configural and metric model CFI diminished with 0.03 while RMSEA increased by 0.012 indicating noninvariance. The metric and scalar model showed nonivariance (CFI measure dropped by 0.07, RMSEA increased with 0.02). These results suggest that direct cultural comparison with the PNI-S should be applied with caution and further research is needed to test the invariance of the construct in these settings.

### Within-subject level analysis

In the multilevel VAR (1) model we used Bayesian estimation based on Markov Chain Monte Carlo chains in Mplus 8.3 (Muthén and Muthén, 1998–2015). We estimated statistical significance by 95% credibility intervals (CI), meaning that each parameter has a 95% chance of falling into this range. If this CI does not contain zero, we can conclude, that the estimate is different from zero (McNeish and Hamaker, 2019).

Our model (DIC = 39129.6; estimated number of parameters: 1207.3) estimates are presented in Figure 1.

Based on the model estimates both the grandiose, both the vulnerable narcissistic state produced significant autoregressive effect on the within subject level. The within-level explained variance (*R*^2^) was 0.04 for the grandiose narcissistic state while 0.14 for the vulnerable narcissistic state.

### Associations on the between-subject level (momentary assessment, Sample 3)

First, to test the association between the PNI-S and state self-esteem, we used repeated-measures correlation (Bakdash and Marusich, 2017). State vulnerable narcissism was in moderate negative association with state self-esteem (*r* = −0.40; *df* = 1,621; *p* < 0.001; CI [−0.44 to −0.36]) while state grandiose narcissism was in a moderate positive association with it (*r* = 0.35; *df* = 1,621; *p* < 0.001; CI [0.31–0.40]). The two state measures of narcissism were in weak negative association (*r* = −0.18; *df* = 1,621; *p* < 0.001; CI [−0.23–0.12]).

Lastly, we tested the associations of the averaged scores of all measurement points of the narcissistic states with other contemporary measures of narcissism and some criterion variables with a set of multiple regression analyses. Grandiose and vulnerable states were used simultaneously as predictors to account for the unique variance each one explains. The results were in line with previous research (see Table 6).

**Table 6:** Associations of the PNI-S in Sample 3: Regression based on averaged scores from the momentary assessments.

	Explained variance (*R*^2^)	Vulnerable narcissistic state (*β*)	Grandiose narcissistic state (*β*)
Narcissistic Personality Inventory (NPI)	0.26	−0.07	**0.49**
Pathological Narcissism Inventory (PNI)	0.20	**0.28**	**0.28**
PNI Grandiosity factors	0.21	0.16	**0.39**
PNI Vulnerability factors	0.15	**0.32**	0.17
Maladaptive Covert Narcissism Scale (MCNS)	0.15	**0.40**	−0.08
Rosenberg Self-Esteem Scale (RSES)	0.23	**−0.35**	**0.42**
State Self-Esteem (SSE)	0.49	**−0.65**	**0.46**
Eudaimonic well-being	0.26	**−0.45**	**0.37**
Gender	0.01	0.01	0.11

*N* = 123. Standardized coefficients. Boldfaced coefficients are significant at least *p* < 0.05. Each covariate is regressed separately to avoid the multicollinearity of covariates. Momentary assessments were used as predictors in the analysis.

State vulnerable narcissism had a medium positive association with other measures of narcissistic vulnerability while state grandiose narcissism had a medium positive association with grandiosity measures. Self-esteem on the trait level and state self-esteem are both negatively associated with state vulnerable narcissism while grandiose narcissism shows positive associations with them.

## Discussion

The present study was designed to develop a state-level measurement tool for grandiose and vulnerable narcissism. As the personality process behind narcissism is gaining more interest, there is a growing need for appropriate measurement tools for capturing momentary fluctuations.

In this study, the *Pathological Narcissism Inventory – State Version (PNI-S)* was first administered in two samples of undergraduates in a cross-sectional design to test the factor structure and between-subject level associations with contemporary measures of narcissism (S1, S2). The two-factor solution yielded an appropriate fit to our data. Vulnerable narcissistic states captured with four items offered higher internal consistency on our first sample, while the factor loadings and the internal consistency of the three-item long state-level grandiose narcissism subscale remained moderate. In S3, we tested the measurement tool in a multilevel confirmatory factor analysis, which provided appropriate fit and factor loadings on both the within-subject, both the between-subject level. These results are in line with our intention, to create a measurement tool as short as possible, not to interfere with the process we study while keeping the reliability and validity of the measure.

State level narcissistic vulnerability and grandiosity were positively associated on the between-subject level, which is in line with previous research using the PNI (Pincus et al., 2009), and it may reflect the entitlement core of narcissism (Wright and Edershile, 2018). The two factors of the PNI-S provided the expected associations with the contemporary measures of trait-level grandiose and vulnerable narcissism and gender. Previous research with the PNI argued that either the grandiosity factor might not be represented sufficiently (Crowe et al., 2019) or vulnerability should be partialed out from the grandiosity construct (Edershile et al., 2019), our results suggest that the PNI-S grandiosity factor is in strong association with other contemporary grandiosity measures (e.g., the NPI, NGS) and weakly or non-significantly associated with measures of vulnerability (NVS, MCNS/HSNS) in both cross-sectional, both ESM designs (for more details, see Tables 1–3, 6). Furthermore, according to our results the nomological network of the PNI-S grandiosity factor is different from the original PNI grandiosity factor especially in associations with narcissistic vulnerability (see Table 3), suggesting that the limited number of state-based items can be effective in partialing out narcissistic vulnerability from the grandiosity factor (Edershile et al., 2019).

Moreover, in accordance with our previous expectations (based on Edershile and Wright, 2021), on the within-subject level the association between vulnerable and grandiose states were negative suggesting that these states are not likely to be present at the same time in individuals. Based on these associations we argue that the PNI-S can sufficiently capture both grandiose both vulnerable narcissistic states and can also differentiate between them.

We also tested the psychometric properties and associations of vulnerable and grandiose narcissistic states on the within-and between person level during a five-day long ESM study. This differentiation is of great importance, because between-subject relations are a good source of information from dispositional, structural variables, that differentiate people from each other, while within-subject associations are offering insights into the internal dynamic process between variables and their dependence on situational factors (Bolger et al., 2003). This measurement tool was specifically designed to enable researchers gathering momentary data in multiple data-collection points.

First, we conducted a multilevel confirmatory factor analysis to examine the fit of the two-factor solution in both the within-and the between-person level. Our results suggest that the scale performs well in both settings, the association between the grandiose and vulnerable states is negative on the within-subject level, while positive on the between-subject level. According to our explanation, vulnerable and grandiose narcissistic states can be associated as overall narcissistic tendencies or traits when we compare individuals on the between-person level (similar to the narcissistic core by Krizan and Herlache, 2018), especially at higher levels of trait grandiose narcissism (Jauk et al., 2022). On the other hand, narcissism – viewed as a self-regulatory function in maintaining a grandiose self (Morf and Rhodewalt, 2001; Back, 2018) – can manifest in different within-person processes (Edershile and Wright, 2021) reflecting on the individual’s specific evaluation of specific situations. This evaluation might result in the unfolding of either grandiose or vulnerable states, and those states are not likely to be present at the same time, although possible shifts between them are suggested by clinical theories (Wink, 1996; Edershile and Wright, 2021). The results from the repeated measures correlation show a similar, somewhat weaker association, where grandiose and vulnerable narcissistic states are negatively associated. It is also worth mentioning, that this rather small effect could not be reproduced in the multilevel Var (1) model in which cross-lagged and autoregressive effects are controlled for. These findings highlight the importance of understanding the narcissistic process itself, not limited to the trait level of narcissism, as everyday functioning might be strongly affected by the internal personality process. This weak negative association should although be investigated further by future research.

Second, the within-person contemporaneous, autoregressive and cross-lagged effects of both vulnerable and grandiose narcissistic states were tested on a multilevel Var (1) model (see Figure 1). Our results suggest that the autoregressive effect (meaning the effect of the preceding narcissistic state on the currently measured one) for both grandiose and vulnerable narcissistic states is meaningful, underlining the stability aspect of these constructs, and their reliability over time (Hamaker et al., 2015). On the other hand, we did not find significant associations between the grandiosity and vulnerability constructs neither on a contemporaneous nor a crossed lagged setting. These results imply that prior states of vulnerability did not predict currently measured grandiosity and vice versa, while current states of grandiosity and vulnerability were also not significantly associated.

The relationship between narcissistic states and self-esteem also served as an important aspect of the validation process of the PNI-S. State level vulnerable narcissism was in medium negative association with both state and trait level self-esteem in all of our analyses which is in line with previous research (e.g., Miller et al., 2010, 2016, 2018). Grandiose narcissism on the other hand has shown non-significant association with self-esteem on a rather trait-like measurement (S1), but moderate positive association on our longitudinal study (S3). On the trait level this weak association is in line with the results of Hyatt et al. (2018) who used a meta-analytic approach and found that the strength of this relationship was between *r* = 0.10 and *r* = 0.43 across different samples. The stronger association on a longitudinal setting on the other hand might highlight the value of capturing the dynamic fluctuations of self-esteem in relation to narcissistic states. At the most general level, these temporal associations suggest that studying narcissism as a fluctuating process consisting of state iterations might enable a deeper understanding of the underlying personality processes of narcissism (Giacomin, 2016; Edershile et al., 2018; Edershile and Wright, 2021).

### Limitations

Despite the strengths of the present study including the different languages of administration, samples, data collection techniques and statistical methods used there are also limitations worth mentioning. The measures used were all self-report measures, and they were administered in student samples overrepresented by women. While this is common practice in current narcissism research, future studies would benefit from collecting informant reports or observing the process of narcissistic personality functioning in laboratory settings and from doing so with clinically diagnosed samples. Furthermore, the administration of other currently available state measures (e.g., the NVS and the NGS) in S3 would have offered more opportunities to study convergent validity, however we aimed to limit the number of items presented to the participants in order to avoid interfering with the internal personality processes we wished to study. Further research with more diverse samples and more balanced gender distributions should also aim to study measurement invariance across genders, cultural settings and age ranges, particularly with the grandiose narcissistic states factor, in which internal consistency remained moderate.

## Conclusion

The present study demonstrated the usefulness of the seven items long *Pathological Narcissism Inventory – State Version (PNI-S).* This measure can perform better than original trait measures of grandiose and vulnerable narcissism (e.g., the NPI or the HSNS) in momentary data collection research where short and current state-related items are crucial in capturing internal states of personality processes. Compared to other currently used momentary measures the PNI-S can be applicable if the entitlement-related core of narcissism is also in focus besides vulnerability and exhibitionism/grandiosity (e.g., the NVS or the NGS) and if vulnerable aspects of narcissistic functioning is equally important in measurement compared to the process-oriented Narcissistic Admiration and Rivalry Questionnaire (Leckelt et al., 2018). Furthermore, our results also highlighted the differences between the within-and between person associations, enabling us to take a closer look into the personality processes behind narcissistic functioning.

## Data availability statement

The raw data supporting the conclusions of this article will be made available by the authors on request.

## Ethics statement

The studies involving human participants were reviewed and approved by Ethical Committee of Psychology, University of Groningen and the Ethical Committee of Eötvös Loránd University, Hungary. The participants provided their written informed consent to participate in this study.

## Author contributions

ME and NR planned and designed the study, collected the data, and wrote the manuscript. ME and RU analysed the data. All authors contributed to the article and approved the submitted version.

## Conflict of interest

The authors declare that the research was conducted in the absence of any commercial or financial relationships that could be construed as a potential conflict of interest.

## Publisher’s note

All claims expressed in this article are solely those of the authors and do not necessarily represent those of their affiliated organizations, or those of the publisher, the editors and the reviewers. Any product that may be evaluated in this article, or claim that may be made by its manufacturer, is not guaranteed or endorsed by the publisher.

## Abbreviations

CFA: confirmatory factor analysis
CFI: comparative fit index
DSEM: dynamic structural equation modelling
TLI: Tucker-Lewis Index
EFA: exploratory factor analysis
ESM: Experience sampling method
MCNS: Maladaptice Covert Narcissism Scale
Multilevel VAR (1): multilevel vector autoregressive model
NPI: Narcissistic Personality Inventory
NGS: Narcissistic Grandiosity Scale
NVS: Narcissistic Vulnerability Scale
PNI: Pathological Narcissism Inventory
PNI-S: Pathological Narcissism Inventory – State Version
SEM: structural equation modelling
S1, S2, S3: Sample 1, Sample 2, Sample 3
